# Supplementary material for: WordCommentsAnalyzer: A windows software tool for qualitative research
Source: F1000Res. 2018 Sep 4;7:536. Originally published 2018 May 3. [Version 2] doi: 10.12688/f1000research.14819.2 (PMC6137411; doi:10.12688/f1000research.14819.2)
Supplement: Supplementary file 3 [file f1000research-7-17614-s0002.tgz › b2a79496-4ada-47c4-9682-a99ce867836d.zip!/Eight Iranian Health Journals/Journal of Research in Rehabilitation Sciences _44.docx]

© Journal of Research in Rehabilitation Sciences . This work is licensed under CC BY-NC 4.0 (https://creativecommons.org/licenses/by-nc/4.0/).

Volume & Issue: دوره 7، شماره 2: 1390

Title: بررسی روایی و اعتبار نسخه ي فارسي آزمون واژه یابی ویرایش دوم در دانش آموزان طبیعی 8-6 ساله ابتدایی

http://jrrs.mui.ac.ir/index.php/jrrs/article/view/151

مقدمه:  یکی از شاخص های مهم که به بررسی و ارزیابی زبان می پردازد توانایی واژه یابی است. ارزیابی های رسمی از مرسوم ترین راه های ارزیابی این توانائی است. آزمون واژه یابی ویرایش دوم یک آزمون رسمی می باشد . با توجه به این که در کشور ما هیچ ابزار رسمی برای ارزیابی این توانایی وجود ندارد، در این مطالعه روایی و اعتبار این آزمون در کودکان 6 تا 8 ساله ی شهر اصفهان تعیین گردید مواد و روش ها: اين مطالعه توصيفي مقطعي بود. آزمون واژه یابی ویرایش دوم پس از ترجمه و تغییرات لازم بر 140 دانش آموزابتدائی که به طور تصادفی انتخاب شده بودند اجرا شد. سپس با استفاده از نظریه ی سوال پاسخ نتایج تحلیل شد  یافته ها: میانگین نمرات واژه یابی و درجه دشواری و قدرت تمیز سوالات آزمون محاسبه ونتایج آن اعلام شد. اعتبار آزمون در فرم ابتدائی برابر با 0.84 بود بحث: میانگین نمرات واژه یابی دانش آموزان فارسی زبان در سطح پایین تری نسب به همتایان انگلیسی زبان قرار دارد و این موضوع می تواند به دلیل بالاتر بودن درجه ی دشواری سوالات آزمون برای دانش آموزان فارسی زبان باشد

Title: بررسي تأثير مهـارت‌هـاي مقـابلـه‌اي شنـاختي- رفتـاري در اضطـراب و سـازگـاري كـودكـان مبتلا به لكـنت زبـان

http://jrrs.mui.ac.ir/index.php/jrrs/article/view/179

مقـدمـه: در دهه‌هاي گذشته، روان‌شناسان روي اثربخشي عوامل خانوادگي، فرهنگي و اجتماعي (به ويژه، رويكردهاي شناختي- رفتاري و مهارت‌هاي زندگي) در سلامت رواني و درمان اختلال‌هاي رواني  تاكيد كرده‌اند.      مـواد و روش‌هـا: اين تحقيق از نوع تحقيق‌هاي نيمه تجربي (طرح پيش‌آزمون- پس‌آزمون و آزمون پيگيري با گروه كنترل) مي‌باشد كه به منظور بررسي تأثير مهارت‌هاي مقابله‌اي شناختي- رفتاري به شيوه گروهي بر كاهش اضطراب و افزايش سازگاري كودكان مبتلا به لكنت زبان در شهر نيشابور صورت گرفته است. بدين منظور 30 نفر از كودكان 8 تا 12 ساله مبتلا به لكنت زبان مراجعه كننده به كلينيك‌هاي گفتار درماني به صورت تصادفي انتخاب و به طور تصادفي به دو گروه آزمايش (15 نفر) و كنترل (15‌نفر) تقسيم شدند. آزمون اضطراب‌اسپنس و سازگاري با لكنت زبان جهت اندازه‌گيري سطح اضطراب و سازگاري آزمودني‌‌ها مورد استفاده قرار گرفت. سپس، آموزش مهارت‌هاي مقابله‌اي شناختي- رفتاري به شيوه گروهي طي 8 جلسه 90 دقيقه‌اي به گروه آزمايش ارائه گرديد، اما گروه كنترل هيچ‌گونه آموزشي را دريافت نكرد. در پايان دوره آموزش و دو ماه بعداز آن، هر دو گروه مجدداً به وسيله آزمون‌هاي فوق مورد ‌آزمون قرار گرفتند.      يـافتـه‌ها: داده‌ها بر اساس آزمونt  در گروه‌هاي مستقل و وابسته و با كمك نرم افزار spss مورد تجزيه و تحليل قرار گرفت. نتايج نشان داد كه آموزش مهارت‌هاي مقابله‌اي شناختي- رفتاري در كاهش اضطراب و افزايش سازگاري با لكنت زبان در آزمودني‌ها مؤثر بوده است (05/0P<).      بـحث: دليل كارآمدي شیوه‌های گروهي‌آموزش‌ مهارت‌هاي مقابله‌اي شناختی-‌‌ رفتاری زیربنای آموزشی آن‌ها است. چرا که در این شیوه هم بر جنبه‌های شناختی و هم رفتاری تاکید می‌شود. از سوي ديگر،  برای اثربخشی بيشتر آموزش‌ها به زمان بیشتری  نیاز است. هم‌چنين، کنترل عوامل اثر‌گذار می‌تواند در اثربخشی بيشتر اين آموزش‌ها موثر باشد.

Title: تاثیر دو، چهار و شش هفته بی تمرینی پس از یک دوره تمرین پلایومتریک بر کنترل پاسچر مردان دانشجو

http://jrrs.mui.ac.ir/index.php/jrrs/article/view/183

مقدمه: کنترل پاسچر يكي از اجزاء اصلی اغلب فعاليت هاي بدنی و فاکتور مهمی براي عملكرد مهارت های ورزشی مي‌باشد. با مروري بر مطالعات گذشته، مشاهده مي شود که نتايج ضد و نقيضي در مورد تاثير برنامه هاي تمريني مختلف و بی تمرینی پس آن بر کنترل پاسچر وجود دارد. لذا هدف مطالعه حاضر بررسی اثر دو، چهار و شش هفته بی تمرینی پس از یک دوره تمرین پلایومتریک بر کنترل پاسچر در مردان دانشجو بود. مواد و روش ها: تعداد 20 نفر دانشجو با میانگین و انحراف استاندارد سنی 35/3 ±2/23 سال، قد 44/5 ±5/171 سانتی متر و وزن 75/7 ±48/74 کیلوگرم بدون سابقه آسیب در دو سال گذشته در تحقیق حاضر شرکت کردند. برای برآورد کنترل پاسچر آزمودنی ها از آزمون [1]SEBT استفاده شد. پس از اجرای پیش آزمون و تشریح نحوه اجرای تمرینات، آزمودنی ها به مدت شش هفته و سه روز در هفته تمرینات پلایومتریک را اجرا کردند. پس از اتمام دوره تمرین پس آزمون اول SEBT اخذ شد. پس از آن آزمودنی ها به مدت شش هفته بدون اجرای تمرینات منظم تحت نظر بودند. در طول دوره بی تمرینی و با فاصله دو هفته ای پس آزمون های بعدی SEBT به عمل آمد. برای تجزیه و تحلیل داده ها از آزمون های آمار توصیفی و آنالیز واریانس با اندازه های تکراری و آزمون تعقیبی توکی (p≤0/05) استفاده شد. یافته ها: نتایج نشان داد که اعمال یک دوره تمرین پلایومتریک تاثیر معنی داری بر افزایش کنترل پاسچر دارد (p≤0/001). هم چنین اعمال دوره بی تمرینی تنها در پس آزمون چهارم (پس از شش هفته بی تمرینی) بر کاهش فاصله دستیابی در آزمون SEBT معنی دار بود (p≤0/01). نتیجه گیری: افزایش کنترل پاسچر پس از دوره تمرین را می توان به افزایش سرعت هدایت عصبی، افزایش قدرت عضلانی و نیز افزایش هماهنگی بین عضلات موافق و مخالف نسبت داد. هم چنین کاهش توانایی کنترل پاسچر پس از دوره بی تمرینی را می توان به کاهش قدرت عضلانی و نیز کاهش هماهنگی عضلانی نسبت داد.

Title: MSبررسي تاثير تمرينات نوروماسكولار بر روي بالانس، راه رفتن و افسردگي بيماران مبتلا به

http://jrrs.mui.ac.ir/index.php/jrrs/article/view/186

مقدمه:مولتيپل اسكلروزيس يكي از شايع ترين بيماري هاي نورولوژيك پيشرونده در افراد جوان بالغ مي باشد، اين بيماري منجر به ايجاد پلاك هاي دميلينه در ماده سفيد دستگاه عصبي مركزي شده و در نتيجه علائم نورولوژيكال را به وجود مي‌آورد. مواد و روش ها: اين مطالعه از نوع آينده نگر، شبه تجربي و يك سويه كور و غير تصادفي است و به منظور بررسي اثر تمرينات نروماسكولار بر روي بالانس، راه رفتن و افسردگي بيماران M.Sدر سال 88- 87در سطح شهر اصفهان انجام گرفته است. در اين مطالعه طي 10  جلسه براي 24 بيمار مبتلا به M.S با شرايط دموگرافي. ( ميانگين سن:06/11  33/33  ، قد: 81/8  66/169    وزن:97/5  87/66 و مدت زمان ابتلا:  19/4  64/6  سال) تمرينات نوروماسكولار به صورت يك روز در ميان انجام شد. اطلاعات حاصل از انجام آزمون ها قبل از درمان ، بعد از درمان و هنگام Follow up جمع آوري گرديد. به منظور بررسي تعادل از Berg Balance scale براي راه رفتن از Dynamic Gait Index و جهت ارزيابي افسردگي از پرسش نامه افسردگي Beck استفاده شد. جهت تجزيه و تحليل آماري نتايج به دست آمده از نرم افزار13 spss و براي مقايسه وضعيت بيماران در مراحل مختلف از آزمون T زوج شده استفاده گرديد. نتايج: پس از بررسي هاي آماري نتايج زير حاصل گرديد: بين وضعيت تعادل، راه رفتن و افسردگی بيماران، قبل و بعد از درمان تفاوت معني داري وجود داشت.(P=0.00) بين وضعيت تعادل P=0.00))وراه رفتن(P=0.03) بیماران قبل از درمان و در  زمان follow up تفاوت معني داري وجود داشت. بين وضعيت تعادل (P=0.00)و راه رفتن(P=0.01) بيماران بعد از درمان و در زمان follow up تفاوت معني داري وجود داشت. بين وضعيت افسردگي بيماران بعد از درمان و در زمان follow up (P=0.14) و  قبل از درمان و در  زمان follow up (P=0.06)تفاوت معني داري وجود نداشت. بحث: نتايج حاصل از اين مطالعه نشان داد كه انجام تمرينات نوروماسكولار به صورت مداوم با فرض ثابت ماندن شرايط بيمار باعث بهبودي در شاخص هاي تعادل، راه رفتن و افسردگي بيماران مبتلا به MSمي شود. با توجه به روند بيماري در صورت توقف انجام تمرينات،  شرايط بيمار به وضعيت اوليه بر خواهد گشت.

Title: شیوع اختلال در بلع دهانی حلقی در میان سالمندان مقیم آسایشگهای سالمندان شهر اصفهان

http://jrrs.mui.ac.ir/index.php/jrrs/article/view/187

مقدمه: اختلال در بلع بویژه در سالمندان می تواند به سوءتغذیه یا یک بیماری ضعیف کننده منجر شود و فرد را از توانایی مراقبت از خویش بازدارد. لذا هدف از انجام این مطالعه بررسی  میزان شیوع اختلال بلع دهانی حلقی در میان سالمندان بستری در آسایشگاه های سالمندان شهر اصفهان بود. مواد و روش ها: این مطالعه توصیفی تحلیلی و از نوع مقطعی بود. تعداد 165 نفر از سالمندان بستری در آسایشگاههای  سالمندان به شکل تصادفی انتخاب شده و پس از مصاحبه تحت آزمون غربالگری اختلال در بلع در سه غظت مختلف مایع، نیمه مایع و جامد قرار گرفتند.سپس نتایج به دست آمده با استفاده از دو آزمون Chi-square و Cochran's Q مورد تجزیه و تحلیل قرار گرفتند. نتایج: شیوع اختلال در بلع در میان افراد سالمند 2/41درصد بود. بعلاوه نسبت بین اختلال در بلع و سه غلظت غذایی نیز مشخص کرد که بیشترین شیوع اختلال در بلع در مایعات می باشد. همچنین شیوع اختلال در بلع در زنان بیشتر از مردان(49% به 31%) بود. علاوه بر این نسبت بین میزان اختلال در بلع در گروههای سنی مختلف نشان داد که با افزایش سن شیوع اختلال در بلع نیز افزایش پیدا می کند. نتیجه گیری: شیوع اختلال در بلع به طور معنا داری در افراد سالمند ساکن خانه های سالمندان شهر اصفهان وجود دارد. آنها با خطراتی نظیر سوء تغذیه و ذات الریه روبرو هستند و از این رونیاز مبرمی به خدمات گفتار درمانی دارند.

Title: مقايسه زاويه سر به جلو در کارکنان مرد مبتلا به گردن درد مزمن و سالم

http://jrrs.mui.ac.ir/index.php/jrrs/article/view/188

هدف: گردن درد مزمن يکي از اختلالات شايع در بين کاربران رايانه مي­باشد. به نظر مي­رسد که افزايش زاويه سر به جلو باعث گردن درد در کاربران رايانه مي­شود. هدف از اين مطالعه مقايسه ميزان سر به جلو در کارکنان مرد مبتلا به گردن درد مزمن و افراد سالم کاربر رايانه دانشگاه اصفهان مي­باشد. روش بررسی: جامعه آماري اين تحقيق شامل 66 نفر مرد کاربر رايانه شامل 33 نفر دچار گردن درد مزمن و 33 نفر سالم مي باشد. براي جمع آوري اطلاعات مربوط به کاربران رايانه از پرسشنامه اطلاعات فردي محقق ساخته، براي اندازه گيري زاويه سر به جلو از گونيامتر و براي سنجش ميزان درد گردن از مقايس بصري درد (VAS) استفاده شده است. . براي تجزيه و تحليل اطلاعات از تي مستقل و ضريب همبستگي پيرسون در سطح )05/0(P<  استفاده شد. یافته ها: ميانگين زاويه سر به جلو در افراد دچار گردن درد مزمن (99/6 ± 97/26) و افراد سالم (19/6 ± 24/24) درجه بود. تفاوت معناداري در ميانگين زاويه سر به جلو هر دو گروه مشاهده شد )05/0(P<. همچنين ميانگين نمره شدت گردن درد در افراد مبتلا به گردن درد مزمن (34/15 ± 7/36 درصد) بود. به طوری که رابطه بين زاويه سر به جلو و ميزان درد گردن معنا دار بود )05/0(P<. نتیجه گیری: با توجه به نتايج پژوهش حاضر مي توان نتيجه گرفت كه افراد مبتلا به گردن درد مزمن نسبت به افراد بدون گردن درد داراي زاويه سر به جلوي بیشتری بودند. و اين مسئله ضرورت توجه به اين افراد و اقدامات پيشگيري کننده، اصلاحي و درماني را بيان مي­نمايد.

Title: Evaluating the effects of a wearable assistive device (WAD) on decreasing lumbar muscles activity during static holding tasks: EMG analysis and biomechanical modeling

http://jrrs.mui.ac.ir/index.php/jrrs/article/view/134

Introduction: One of the most common diseases associated with musculo-skeletal ailment is low back pain which is epidemic among general public. If manual tasks are performed inappropriately by workers at workplaces, it may cause back pain in the lumbar spine. In recent years, a variety of assistive devices has been developed and employed to utilize a passive force generator which has been proved to be effective in decreasing lumbar moments and the force requirements by Erector Spinae muscles. In forward flexion, trunk muscles sustain a balance between the weight of upper body and external loads. Because of small moment arm of these muscles, they can create great forces that may lead to high intradiscal pressure and cause disc injury. The aim of this paper is to present a new Wearable Assistive Device that decreases trunk muscle activity during static holding task. The performance of this device is simulated biomechanically with link-segment model. Materials and Methods: Using ANYBODY software, compressive and shearing forces and also muscle activity of Lumbar were determined. To validate the results, electromyography (EMG) of fifteen males’ muscles participated in the study was analyzed.  Results: Results of link-segment model indicated that there was a reduction in the Lumbar moment by 15- 23% using WAD. Moreover ANYBODY model demonstrated that compressive and shearing forces decreased by 24% and 29% respectively in average and muscle activities decreased by 18-25%, too. Statistical analysis indicated that normalized electromyography of right and left Lumbar, Thoracic Erector Spinae and Latissimus Dorsi muscles had significantly decreased  by 21.9%, 20.4%, 23.6%, 20.4%, 23.7%, and 16.7% (p<0.05) respectively and electromyography of right and left External Oblique, Internal Oblique and Rectus Abdominus muscles had underwent a significant change. (p<0.05)   Keywords: Wearable Assistive Device (WAD), Lumbar moments, Muscle activity, Biomechanical model, EMG

Title: مقايسه تاثير دو روش تمرين درماني و خود درماني از طريق "کتاب کمر"بر بيماران مبتلا به کمردرد مزمن

http://jrrs.mui.ac.ir/index.php/jrrs/article/view/215

مقدمه: با توجه به تنوع شيوه هاي درماني براي کمردرد ، هنوز توافق نظر درباره ي موثرترين روش درماني وجود ندارد. هدف از انجام اين پژوهش، مقايسه برنامه تمريني منتخب و درمان با نگرش خود درماني با استفاده از کتابي نوين در درمان کمردرد با عنوان"کتاب کمر"[1] مي باشد. مواد و روش ها: 29 بيمار با تشخيص کمر درد مزمن در اين پژوهش شرکت کردند که به صورت تصادفي در دو گروه تمرين درماني (17بيمار) و خود درماني بوسيله" کتاب کمر" (12 بيمار) قرار گرفتند. گروه تمرين درماني با تشخيص پزشک تمرينات منتخب فلکسوري، اکتنسوري و تمرينات ثبات کمر را دريافت کردند و گروه خود درماني ملزم به خواندن "کتاب کمر" و رعايت مفاد آن گرديدند. طول مدت درمان براي هر دو گروه 6 هفته در نظر گرفته شد. ميزان درد و ناتواني به ترتيب بوسيله  شاخص بصري درد وس[2] و پرسشنامه ناتواني عملکردي اوسوستري[3] ارزيابي شد. يافته ها با استفاده از آزمون تي زوجي و تي مستقل در سطح معناداري  05/0 تحليل شد. نتايج: نتايج اين مطالعه نشان داد که تمرين درماني باعث کاهش معنادار در ميزان درد )001/0 (p<وبهبود ناتواني)001/0= (p  بيماران گروه تمرين شده است در حاليکه گروه " کتاب کمر" در دو متغير درد و ناتواني بهبود معناداري را نسبت به قبل از دوره درمان نشان ندادند) 05/0 (p>. همچنين مقايسه بين دو گروه براي متغيرهاي درد و ناتواني تفاوت معناداري را از نظر آماري با يکديگر نشان دادند. بحث: تمرين درماني روشي موثر در درمان کمردرد مزمن مي­باشد. همچنين نتايج تحقيق نشان داد که تمرين درماني بيش از خوددرماني به وسيله "کتاب کمر" در درمان کمردرد مزمن تاثير دارد. از اين رو به نظر مي­رسد تمرين درماني به اين شکل بتواند روشي مؤثر در درمان کمردرد مزمن به شمار رود.

Title: رابطه بين ناهنجاري زانوي پرانتزي با عملكرد و قدرت اندام تحتاني در فوتبالیست های نوجوان

http://jrrs.mui.ac.ir/index.php/jrrs/article/view/216

مقدمه: هدف پژوهش حاضر بررسی رابطه بین ناهنجاری زانوی پرانتزی با عملكرد و قدرت اندام تحتاني در فوتبالیست­های نوجوان بود. مواد و روش­ها: برای انجام اين تحقيق 60 فوتباليست نوجوان با میانگین سنی78/0 ±2/15 سال، میانگین وزنی39/3±9/58 کیلوگرم و میانگین قد21/6±166 سانتی­متر انتخاب شدند. براي ارزيابي وضعيت بدني آزمودني­ها از روش كندال و خط شاقول استفاده شد. ناهنجاري زانوي پرانتزي با استفاده از كوليس اندازه­گيري و ثبت شد. همچنین براي بررسي قدرت و عملکرد اندام تحتانی آزمودني­ها از آزمون اسكوات روي يك پا و بدست آوردن حداكثر فاصله، بيشترين فاصله بدست آمده از لي­لي روي يك پا، انجام سه لي­لي متوالي روي يك پا و بدست­آوردن بيشترين فاصله، پرش عمودي و آزمون­تي استفاده شد. برای بررسی اختلاف آزمون­ها در دو گروه تحقیقی از تی­مستقل استفاده شد. نتایج: با توجه به نتایج بدست آمده از تحقيق حاضر، اختلاف معنی­داری بین دوگروه در اجرای آزمون­ها یافت نشد[پرش عمودی(P=0.005)، اسكوات روي يك پا و بدست آوردن حداكثر فاصله(P=0.019)، بيشترين فاصله بدست آمده از لي­لي روي يك پا (P=0.011)، انجام سه لي­لي متوالي روي يك پا و بدست آوردن بيشترين فاصله (P=0.008) و آزمون تي (P=0.003)]. بحث و نتیجه­گیری: با توجه به نتایج بدست آمده از این تحقیق نتیجه­گیری می­شود وضعيت بدني در متغيرهاي اجرا در فوتباليست­ها تاثیرگذار بوده و ناهنجاري زانوي پرانتزي مي­تواند باعث تضعيف اجرا گردد.

Title: Comparing bone mineral density (BMD) of spinal L2-L4 in Iranian swimmers and their foreign counterparts

http://jrrs.mui.ac.ir/index.php/jrrs/article/view/147

Introduction: It has been shown through various studies that athletes who compete in such non–weight-bearing sports as swimming typically show lower BMD in the lumbar spine and lower limb sites than those who are involved in weight-bearing sports. Gravitational forces produce a passive mechanical effect that plays a role in developing and maintaining of bone mass. This study was conducted with the aim of comparing the bone mineral density (BMD) of spinal L2-L4 in Iranian swimmers with that of their foreign counterparts. Materials and Methods: Twelve professional male swimmers (mean age 24±3.3 yr, mean weight 81±1.1 kg, and mean height 175±5.2 cm) were compared with fourteen  professional males foreign swimmers (mean age 22±3.6 y, mean weight 72±10 kg, mean height 182±7.2 cm). These subjects, all in good health, were randomly selected from the available population of athletes who had been involved in continuous swimming activities for past five years. BMD were measured on L2-L4 lumbar spines via dual-energy X-ray absorptiometry (DEXA).The data were analyzed through one –sample mean comparison and Pierson correlation.  Results: The results showed that the amount of BMD in Iranian swimmers (1.02 ± 0.04 g.cm²) was less than that of foreign swimmers (1.16 ± 0.16 g.cm² ، (p=0.000)). Conclusion: Considering the importance of BMD in lumbar spine for swimmers and its significant loss among Iranian swimmers compared to foreign swimmers, which was evident from the results obtained, it is necessary that swimming must be accompanied by weight-bearing exercises and a diet rich in dairy. Key Word: Bone mineral density, Dual-energy X-ray absorptiometry, Lumbar spine, Swimming, Osteoporosis

Title: تاثير چهار هفته تمرين درماني بر زاويه Q و Popliteal در ورزشكاران مبتلا به سندرم درد كشككي- راني

http://jrrs.mui.ac.ir/index.php/jrrs/article/view/217

مقدمه و هدف: سندرم درد كشككي راني واژه كلينيكي شايعي جهت توصيف شرايط پاتولوژيك درد موجود بين کشکک و كنديل هاي ران است(لطافت­کار و همکاران 2010[1]).  هدف از انجام این تحقیق بررسی تاثير چهار هفته تمرين درماني بر زاويه Q و Popliteal در ورزشكاران مبتلا به سندرم درد كشككي- راني بود. روش کار: تحقیق نیمه تجربی حاضر بر روي 60 ورزشکار مبتلا به درد کشککي- راني در رشته­های والیبال، کاراته و تکواندو با میانگین سن 15/0± 38/21 سال، قد 52/1± 18/ 170 سانتی­متر و وزن 67/0± 79/60 کیلوگرم و سابقه ورزشی آنها بیش از 3 سال در دو گروه 30 نفري تجربی و کنترل انجام شده است. در اين تحقيق زاويه Q و پوپليتئال با استفاده از گونيامتر و شدت درد زانو با مقياس بصری درد (VAS) قبل و بعد از تمرين­درماني چهار هفته­اي سنجيده شد. داده­های بدست آمده از طریق آزمون­های آماري تی­زوجی و  تی­مستقل در نرم افزار Spss ورژن 17 مورد تجزیه و تحلیل قرار گرفت(سطح معنی­داری 05/0). یافته­ها: یافته­ها نشان داد که چهار هفته تمرين درماني، تاثیر معنی­داری در کاهش میزان درد زانوی ورزشکاران دارای سندرم درد کشککی رانی دارد. همچنین انعطاف­پذیری عضلات همسترینگ و قدرت عضله چهارسر رانی نیز بعد از اجرای برنامه حرکت درمانی افزایش یافت. یحث و نتیجه­گیری: نتایج این تحقیق نشان داد که برنامه های تمرینی کششی و تقویتی در عضلات خم و راست کننده زانو ممکن است در درمان سندرم درد کشککی رانی موثر باشد. اثبات این مورد نیازمند انجام تحقیقات بیشتری می­باشد.

Title: بررسي تأثير شش هفته تمرینات قدرتي و پلايومتريک بر تعادل پوياي دانشجويان مرد ورزشکار

http://jrrs.mui.ac.ir/index.php/jrrs/article/view/228

مقدمه:‌ تعادل از اجزاء جداناپذير تقريباً همه فعاليت‌هاي روزانه و شاخصي تعيين کننده در بررسي توانايي عملکردي ورزشکاران به شمار مي رود. بنابراين هدف از انجام تحقيق حاضر بررسي تأثير شش هفته تمرينات پلايومتريک و ترکيبي بر تعادل پوياي دانشجويان مرد ورزشکار بود. مواد و روش‌ها: آزمودني‌هاي تحقيق حاض را30 دانشجوي مرد ورزشكار با ميانگين سني 18/23 سال، وزن 17/72 کيلوگرم و قد 58/173 سانتي‌متر تشكيل دادند كه بدون سابقه آسيب در اندام‌ها بوده و به صورت داوطلبانه در اين تحقيق شرکت و به صورت تصادفي به دو گروه 15 نفري]گروه يک: تمرين ترکيبي(قدرتي و پلايومتريک) و گروه دو: کنترل[ تقسيم شدند. قبل از اعمال شش هفته برنامه تمريني، تعادل پوياي آزمودني‌ها به وسيله آزمون تعادل ستاره SEBT))[1] اندازه گيري شد. در طول شش هفته که گروه تمريني، تمرينات ويژه خود را انجام مي‌دادند، گروه کنترل نيز به فعاليت‌هاي بدني روزانه و تمرينات خود پرداختند. بعد از اتمام دوره تمريني، تعادل پوياي آزمودني­ها نيز اندازه‌گيري شد. از آمار توصيفي براي محاسبه‌ي ميانگين ويژگي‌هاي آزمودني­ها و نيز فاصله دستيابي آنها در هشت جهت SEBT، استفاده شد. همچنين از آزمون تي مستقل براي مقايسه فاصله دستيابي آزمودني­ها در دو گروه پس از اعمال برنامه تمريني و از آزمون تي همبسته براي تعيين تأثير تمرينات بر تعادل پويا، در سطح معني داري 05/0 α< استفاده شد. يافته ها: پس از اجراي دوره تمريني و در پس آزمون SEBT فاصله دستيابي آزمودني­هاي گروه تمريني در تمامي جهت ها افزايش معني داري داشت. اما در فاصله دستيابي آزمودني‌هاي گروه کنترل در پيش و پس آزمون SEBT هيچ تفاوت معني‌داري مشاهده نشد. همچنین پس از اعمال برنامه تمرینی بین گروه تمرینی و کنترل در تمامی جهت ها تفاوت معنی داری مشاهده شد. نتيجه گيري: با توجه به نتايج تحقيق استفاده از تمرينات ترکيبي(پلايومتريک و قدرتي) به منظور کاهش احتمال آسيب و بهبود تعادل پوياي ورزشکاران توصيه مي­شود.

Title: آسیب کاربرد شناختی زبان: پژوهش موردی

http://jrrs.mui.ac.ir/index.php/jrrs/article/view/240

مقدمه: کودکان دارای آسیب کاربردشناختی زبان در کاربرد زبان متناسب با سن و موقعیت مشکل دارند. در مطالعه ی حاضر به ارزیابی و درمان یک کودک دارای آسیب کاربرد شناختی زبان پرداخته شده است. مواد و روش ها: در این مطالعه از روش جمع آوری اطلاعات مشاهده ای استفاده شد. سپس دو فرم نیمرخ شریک ارتباطی و مقیاس نمره دهی مهارت های کاربرد شناختی کودک تکمیل شد. با توجه به نتایج حاصل از دو فرم، مداخله ی درمانی مناسب انتخاب شد. یافته ها: پس از ارائه ی درمان و مشاورات به والدین جهت بهبودی مهارت های مکالمه ای و کاربرد کنش های گفتاری، نمره ی کودک در مقیاس نمره دهی کاربردشناختی به طور معناداری افزایش یافت. همچنین توانائی کودک در مهارتهای مکالمه ای به طور متوسط 3-2 نمره افزایش یافت. بحث: درمان انتخاب شده در این مطالعه شبیه مطالعه ی ریچاردسون و کلیکان- اکر، متمرکز بر مهارت های کاربردشناختی زبان بود. این جنبه ی زبانی به عنوان یک کل در نظر گرفته شد نه به عنوان بخشی ازدرمان.در نتیجه، تشخیص مناسب اختلال در تصمیم گیری جهت ارزیابی و انتخاب برنامه ی درمانی مناسب مؤثر است.

Title: اختلال پردازش شنیداری (مرکزی) درآسیب شناسی گفتار و زبان

http://jrrs.mui.ac.ir/index.php/jrrs/article/view/214

مقدمه: مهارتهای پردازش شنیداری پایه شنیدن ، ارتباط و یادگیری را فراهم می کنند و نقایص مربوط به آن باعث اشکالات ارتباطی و خصوصاً مشکلات تحصیلی در کودکان می شود. آگاهی از علایم این اختلال، روند ارزیابی و مداخلات درمانی مناسب در حیطه مسئولیت های آسیب شناسان گفتار و زبان و شنوایی شناسان است و عدم آگاهی نسبت به وظایف حرفه ای   باعث سردرگمی در تشخیص و درمان ناکارآمد می شود. هدف از این مقاله پاسخ دهی به تعدادی از سوالات رایج آسیب شناسان گفتار و زبان در زمینه ماهیت، تشخیص و درمان APD (C) وهمچنین جلوگیری از استفاده نادرست از این اصطلاح می باشد. به علاوه سعی می شود مسئولیتهای حرفه ای آسیب شناسان گفتار و زبان نیز در این زمینه تبیین شود. روش: این مقاله مروری است بر آخرین مقالات و کتابهای منتشر شده در زمینه اختلال پردازش شنیداری (مرکزی) که در پایگاههای اطلاعاتی PUBMED, EBSCO, SCIENCE DIRECT , ASHA , GOOGLE SCHOLAR THIEME   PROQUEST, و همچنین در منابع کتابخانه ای مورد جستجو قرار گرفت. نتیجه گیری:  نتایج این بررسی حاکی از آن است که پردازش شنیداری فرآیندی پیچیده است و آسیب شناسان گفتار و زبان در زمینه غربالگری، تشخیص افتراقی و درمان افراد مبتلا به  (C)APDنقش مهمی را به عهده دارند و این متخصصان خصوصاً در هنگام وجود مشکلات یادگیری و یا توجه در کودکان مدرسه رو بایستی احتمال وجود APD(C) را مد نظر قرار دهند.
